# Living with psychosis and cognitive impairment: a qualitative study

**DOI:** 10.1186/s12888-026-07861-0

**Published:** 2026-02-02

**Authors:** Tobias Landström, Anna Björkdahl, Mårten J. Tyrberg, Maria Lindau

**Affiliations:** 1 Centre for Clinical Research Västmanland, Uppsala University, Västerås, Sweden; 2 Centre for Psychiatry Research, Department of Clinical Neuroscience, Karolinska Institutet, & Stockholm Health Care Services, Region Stockholm, Sweden; 3 Department of Psychology, Uppsala University, Uppsala, Sweden; 4 Department of Psychology, Stockholm University, Stockholm, Sweden

**Keywords:** Psychotic spectrum disorder, Schizophrenia, Cognition, First person experience, Lived experience, Functioning

## Abstract

**Background:**

Cognitive impairment is a major contributor to functional disability in individuals with psychosis. However, there is limited qualitative research on the everyday experience of cognitive impairments in psychosis. Addressing this gap could inform more person-centered care. This study aimed to gain a deeper understanding of how cognitive impairments affect daily life among individuals living with psychosis.

**Methods:**

A qualitative phenomenological approach was employed. Data was collected through semi-structured individual interviews with nine participants, aged 30 to 55 years, diagnosed within the psychosis spectrum. Inclusion required evidence of cognitive impairment, operationalized as scoring at least one standard deviation below the normative mean on one or more cognitive domains assessed by the Repeatable Battery for the Assessment of Neuropsychological Status (RBANS). Interviews were transcribed and analyzed using thematic analysis.

**Results:**

Four main themes emerged: (1) Acknowledging and making sense: participants recognized diverse manifestations of cognitive difficulties in their daily life and sought to interpret and contextualize them in order to make sense of their experiences; (2) Navigating the importance and challenges of social life: social interactions were valued but often hindered by sensory overload and feelings of loneliness, and were mostly limited to family; (3) Adjusting to a slow pace: participants described a daily life marked by passivity and difficulties in planning and structuring meaningful activities, leading to lowered expectations for activities and stimulation; and (4) Using everyday workable strategies: coping with cognitive challenges including choosing less crowded environments, engaging in activities requiring short periods of concentration, as well as adopting an accepting attitude towards life.

**Conclusions:**

By exploring participants’ lived experiences of cognitive difficulties, this study highlights not only the heterogeneity in how such impairments are manifested, but also the varying degrees of awareness with which they are understood. These insights support the need for individually tailored approaches, both in adapting treatments such as cognitive remediation and in developing interventions that empower individuals to enhance their awareness of their challenges and support strength-based coping strategies.

**Trial registration:**

Clinical trial number: not applicable.

## Background

Cognitive impairment has been identified as a core feature of psychotic spectrum disorders [1], affecting the majority of individuals across the psychotic spectrum [2, 3] and significantly impacting overall functioning [4]. People with psychotic spectrum disorders consistently perform worse than healthy controls on neuropsychological tests across most cognitive domains [2, 5], such as working memory, problem-solving, and social cognition [6]. Although severity varies, cognitive deficits tend to be greatest in schizophrenia [7] but are also present in schizoaffective disorder [8]. Evidence in delusional disorder is more limited and findings to date are mixed [9, 10]. Cognitive impairments in psychotic spectrum disorders are evident before the initiation of antipsychotic drugs [11], and longitudinal studies suggest their presence prior to the onset of psychotic symptom [12, 13]. Additional studies indicate that cognitive impairments often remain stable from the prodromal to the early illness phase and do not appear to worsen with the emergence of psychotic symptoms [14–17]. However, the overall trajectory remains unclear, with other studies reporting declines in IQ and a range of other cognitive functions during the early course of illness [18]. While a substantial body of research underscores the centrality of cognitive impairment in psychotic spectrum disorders, available treatments have so far shown only modest efficacy. Some studies have reported cognitive benefits from antipsychotic treatment [19]; however, later evidence suggests that any improvements in cognitive performance are at best small [20], and concerns have also been raised regarding potential adverse effects on cognitive functioning [21]. Cognitive remediation, a behavioral training based intervention aimed at improving cognitive processes such as attention and memory, has shown small to moderate efficacy in clinical trials [22–24]. However, despite this promising evidence, real-world implementation remains limited, partly due to barriers such as accessibility, feasibility and lack of routine integration into standard care [25, 26]. As a result, many individuals continue to struggle with cognitive difficulties in daily life, sometimes without having discussed these challenges with their treatment team [27]. This is particularly concerning given that people with schizophrenia often prioritize cognitive difficulties over positive symptoms when discussing treatment needs [28].

More knowledge is needed to understand how cognitive interventions can be better tailored to individuals’ lived experiences and everyday needs. Although quantitative research has provided valuable insights into the prevalence and severity of cognitive impairments, it often fails to capture phenomenological aspects such as the full scope of lived experience, meaning how people live through and make sense of their experiences in their everyday lives [29]. Similarly, standardized neuropsychological tests typically focus on specific cognitive domains, but do not always reflect the everyday challenges that people encounter [30]. This underreporting of first-person perspectives is problematic given the complexity of psychosis as a condition that fundamentally affects the sense of self, perception, and cognitive processes [31]. Qualitative approaches have the potential to fill this research gap by capturing the richness that first-person accounts offer. A growing body of research has already demonstrated the value of incorporating lived experience in understanding key features of psychosis, including delusions [32] and the early stages of psychotic illness [33]. However, despite the centrality of cognitive impairment to psychosis, only a few studies have explored how individuals themselves experience and narrate these difficulties [31].

In one such study, the lived experiences of cognitive impairments in eight men with schizophrenia were explored using qualitative methods. The authors found that participants’ difficulties appeared to stem from an inability to direct cognitive resources, rather than from a cognitive deficit per se [34]. In a similar study, ten young adult participants, each in an early phase of psychosis, were individually interviewed about their cognitive functioning and its relationship with psychosocial functioning. Some participants reported difficulty distinguishing cognitive impairments from psychiatric symptoms. The study also found that participants had developed self-generated coping strategies for managing cognitive challenges, such as using repetition to support memory [35]. Such accounts have been suggested to offer a more nuanced perspective by shifting attention from deficits to cognitive strengths, and by highlighting not only impairments but also preserved or adaptive abilities that may support self-efficacy [27, 36].

These studies have initiated a language and conceptual framework for describing the lived experience of cognitive impairment in psychosis. However, a recent review of qualitative studies on cognitive health in psychosis underscored not only a general scarcity of such research, but also pointed to methodological weaknesses. These included inconsistent reporting of participant characteristics and the lack of involvement of service users in the planning of the studies [31]. Given these gaps, further research is needed to enhance our understanding of how cognitive impairments are experienced in psychosis. Such knowledge could inform not only the development of more personalized treatment approaches, but also the adaptation of interventions to better match the lived experiences of individuals with psychosis. By aligning cognitive remediation with the ways individuals perceive and manage their cognitive challenges, acceptability and engagement may be increased, helping to overcome barriers related to accessibility.

In the current study, the aim was to gain a deeper understanding of lived experiences of individuals with psychosis regarding cognitive impairments and their impact on daily life. Specifically, the study sought to answer the following research question: How do people with psychosis describe what it is like to live with cognitive impairment?

## Methods

### Design

A qualitative phenomenological research approach was used, based on individual interviews and inductive thematic analysis. A cognitive assessment was included to both verify eligibility (i.e., presence of cognitive impairment) and provide descriptive information relevant for interpreting the participants’ lived experiences.

### Participants

Participants were recruited between April and September 2024 from a psychiatric outpatient unit specialized in psychosis, located in a medium-sized city in central Sweden with approximately 160 000 inhabitants. Inclusion criteria were a diagnosis of a primary psychotic spectrum disorder, age between 18 and 55 years (the upper limit was chosen to reduce the influence of age-related cognitive decline unrelated to psychosis), evidence of cognitive impairment as indicated by a score of at least one standard deviation below the normative mean on one or more of the five subscales of the Repeatable Battery for the Assessment of Neuropsychological Status (RBANS) [37], and adequate spoken Swedish, assessed pragmatically by confirming that participants could understand questions and communicate their experiences sufficiently to participate in the interview. Diagnostic information was obtained from participants’ medical records, where diagnoses are established by treating psychiatrists according to ICD-10 criteria. Exclusion criteria included high levels of distress due to current psychotic symptoms or recent life events (as assessed by the individual and their treating clinician). Distress was defined pragmatically as a level of emotional or symptom-related strain that would make it difficult for the person to complete cognitive testing or participate meaningfully in an interview. Additional exclusion criteria included evidence of intellectual disability (IQ < 70), or any current or prior contact with the research leader (author TL, an experienced clinical psychologist), which could influence the decision to participate. The study was introduced during a staff meeting at the clinic, where the need to recruit participants was emphasized. The staff were encouraged to approach all patients who met the inclusion criteria, rather than only those suspected of having cognitive impairments. Patients who expressed interest received both written and verbal information about the study from their treating clinicians and provided written informed consent.

### Procedures

Once a participant agreed to take part, the research leader was informed and contacted the individual to schedule a session for neuropsychological testing using RBANS. The test lasted approximately 20–30 min. After testing, each participant was contacted to receive feedback on their results. Individuals who scored at least one standard deviation below the normative mean on one or more of the five subscales of the RBANS were invited to a second session for a qualitative interview with the research leader. In total, 31 individuals were approached for participation; 19 expressed interest and were subsequently contacted. However, some could not be reached or experienced a worsening of symptoms. Twelve participants completed the cognitive assessment, of whom nine proceeded to the interview stage. One participant performed within the normal range across all five RBANS subscales and was therefore excluded. Another participant found the testing too demanding and withdrew during the session, while one additional participant chose to cancel participation prior to the interview. The final sample included nine participants (see Table 1 for demographic details). All participants were in a clinically stable phase as judged by their treating clinicians, who confirmed that they were not experiencing acute psychotic symptoms at the time of recruitment. Participants themselves also reported being able to take part in an interview without undue distress.

**Table 1:** Demographic and clinical characteristics of study participants

Variable	Category / Statistic	Value
Gender	Male / Female	6 / 3
Age	Range (years)	30–55
	Mean (SD)	42.7 (9.8)
Diagnoses	Schizophrenia	4
	Schizoaffective syndrome	2
	Unspecified nonorganic psychosis	3
Education	Range (years)	8–16
	Mean (SD)	13.1 (2.5)
Duration of psychosis treatment	Range (years)	2–40
	Mean (SD)	18.3 (10.3)
Antipsychotic medication	Taking regularly	8
Employment status	Sick leave	3
	Disability pension	4
	Working	2
Global RBANS Index score*	Range	69–91
	Mean (SD)	79.8 (10.4)

* Global RBANS Index Score was available for 8 of the 9 participants. For one participant, a global score could not be calculated because one subtest could not be completed according to standard procedures. This did not affect inclusion, as the participant had valid subscale scores meeting the inclusion criterion

### Neuropsychological testing

RBANS is a standardized screening tool designed to evaluate a broad range of cognitive functions. It comprises 12 subtests, grouped into five index scores: *Immediate memory*,* Visuospatial/Constructional ability*,* Language*,* Attention* and *Delayed memory.* Each index score is standardized against a general population sample, with a mean of 100 and a standard deviation of 15. A total scale index score provides a summary of overall cognitive performance. RBANS was selected for this study due to its demonstrated sensitivity to cognitive impairment in individuals with schizophrenia [38], as well as its relatively brief administration time (approximately 25 min). Because the purpose of the assessment was to determine the presence of cognitive impairment, participants’ scores were compared to normative data for healthy controls. Impairment, as defined by the study’s inclusion criteria, was indicated by scoring at least one standard deviation below the normative mean on one or more of the five RBANS domains. This cut-off was chosen to ensure the inclusion of participants with cognitive deficits, including those with milder impairments. A lower threshold risked excluding participants who could meaningfully engage in the interview process. The RBANS was administered in person by the research leader, using the Swedish paper-and-pencil version.

### Interview protocol

Previous qualitative studies have directly asked participants about cognitive difficulties [27, 34, 35], which, while valuable, may limit the breadth of inquiry. Cognitive impairments can be overlooked or misattributed to general symptoms of psychosis or overall malaise [34, 35]. As a result, participants may not always recognize or articulate certain cognitive difficulties, or they may overlook more subtle aspects of cognitive impairment that nontheless affect daily functioning. In this study, a more open-ended interview approach was employed, aimed at exploring participants’ broader experiences of daily life. While the interviewer kept cognitive themes in mind, the questions were not limited to or explicitly framed around cognitive difficulties.

A semi-structured interview guide was developed based on a lifeworld phenomenological approach [39] and the specific aim of the study. Participants were first invited to describe a typical day—how it begins, what they do, and how it unfolds. They were then asked to reflect on what characterizes a good versus a bad day. When participants described challenges that the interviewer (author TL) interpreted as potentially related to cognitive functioning, they were encouraged to elaborate and reflect on how these experiences influenced their everyday interactions and activities.

To ensure relevance and clarity, the interview guide was piloted with an individual with lived experience of psychosis and cognitive impairment, recruited through a patient advocacy organization. Following the pilot interview, the individual provided feedback on the guide’s content, structure, and wording, which informed subsequent refinements. All interviews were conducted in Swedish and lasted between 16 and 46 min. They were conducted, transcribed, and translated by the author TL.

### Data analysis

Data were analyzed using thematic analysis, following the six phases outlined by Braun & Clarke [40]. An inductive, data-driven approach was employed to capture the participants’ lived experiences of cognitive impairment without posing a pre-existing coding frame. Thematic analysis was chosen for its flexibility and suitability for capturing patterns of meaning in under-researched areas. In contrast to deductive, hypothesis-driven approaches, an inductive method allowed us to explore how individuals make sense of cognitive difficulties in ways not always captured by standardized neuropsychological assessment or previous quantitative research. While the analysis was primarily inductive and data-driven, the semi-structured interview guide prompted participants to elaborate on potential cognitive difficulties when these arose naturally. Thus, the analytic approach may be considered a pragmatic combination of inductive coding with mild deductive guidance from the study’s focus on cognition.

The analysis was led by the first author (TL), who conducted Phases 1 to 3. TL began by familiarizing themselves with the data through reading and transcribing the interviews (Phase 1). The interviews were manually transcribed verbatim by TL using Microsoft Word. No formal verification procedures (e.g., second-researcher cross-checking) were conducted, but transcription accuracy was supported through repeated readings during the familiarization phase. This was followed by a complete coding of the dataset (Phase 2), in which meaningful features relevant to the research question were labeled. Coding was semantic in nature, focusing on the explicit content of participants’ accounts rather than trying to interpret underlying meanings or latent constructs.

As TL is a clinical psychologist with extensive experience working with individuals with psychosis, this professional background may have shaped initial expectations during coding. To address this, reflexive notes were kept throughout the analytic process, and preliminary interpretations were regularly discussed with co-authors to challenge assumptions. In Phase 3, TL identified patterns among the codes and developed potential themes to capture important aspects of the data in relation to the research question. In Phase 4, the themes were reviewed and refined through an iterative process, involving TL in collaboration with co-authors AB and ML. This stage included revisiting both the coded data and the full dataset. Consensus and saturation were reached through repeated discussions and adjustments. In Phase 5, the themes were defined and named collaboratively by TL, AB and ML. Theme names were developed through a process of abstraction, aiming to capture the underlying pattern across multiple code clusters, while subthemes were kept close to the participants’ language to reflect more specific aspects withing each thematic area. In the final phase (Phase 6), the themes were written up in a coherent narrative that forms the basis of the findings. All coding was done manually using Microsoft Word processing software.

### Ethical considerations

The research was conducted in accordance with the Declaration of Helsinki. Ethics approval was obtained from the Swedish Ethical Review Authority (reference number 2023-07757-01). To protect participant confidentiality, individual cognitive test results are not presented in this report.

### Trustworthiness aspects

To establish the trustworthiness of the study, several aspects were addressed. To enhance transferability, detailed demographic data were provided, including assessments of cognitive impairment. Throughout the analysis, the authors managed the risk of bias through reflexive practice, critically examining their preconceptions, perspectives, and positionality, which could potentially influence data interpretation [41]. This was particularly relevant for author TL, who led the research and has extensive experience in assessing and treating individuals with psychosis and cognitive impairment. Furthermore, to strengthen credibility and transparency, participants’ quotes were incorporated when reporting the results. In addition, the relevance and accessibility of the data collection process were enhanced through the involvement of a person with lived experience of psychosis and cognitive impairment in the development of the interview guide. This participatory step improved the guide’s sensitivity and ensured that the questions resonated with the target population.

## Results

**Fig. 1:**
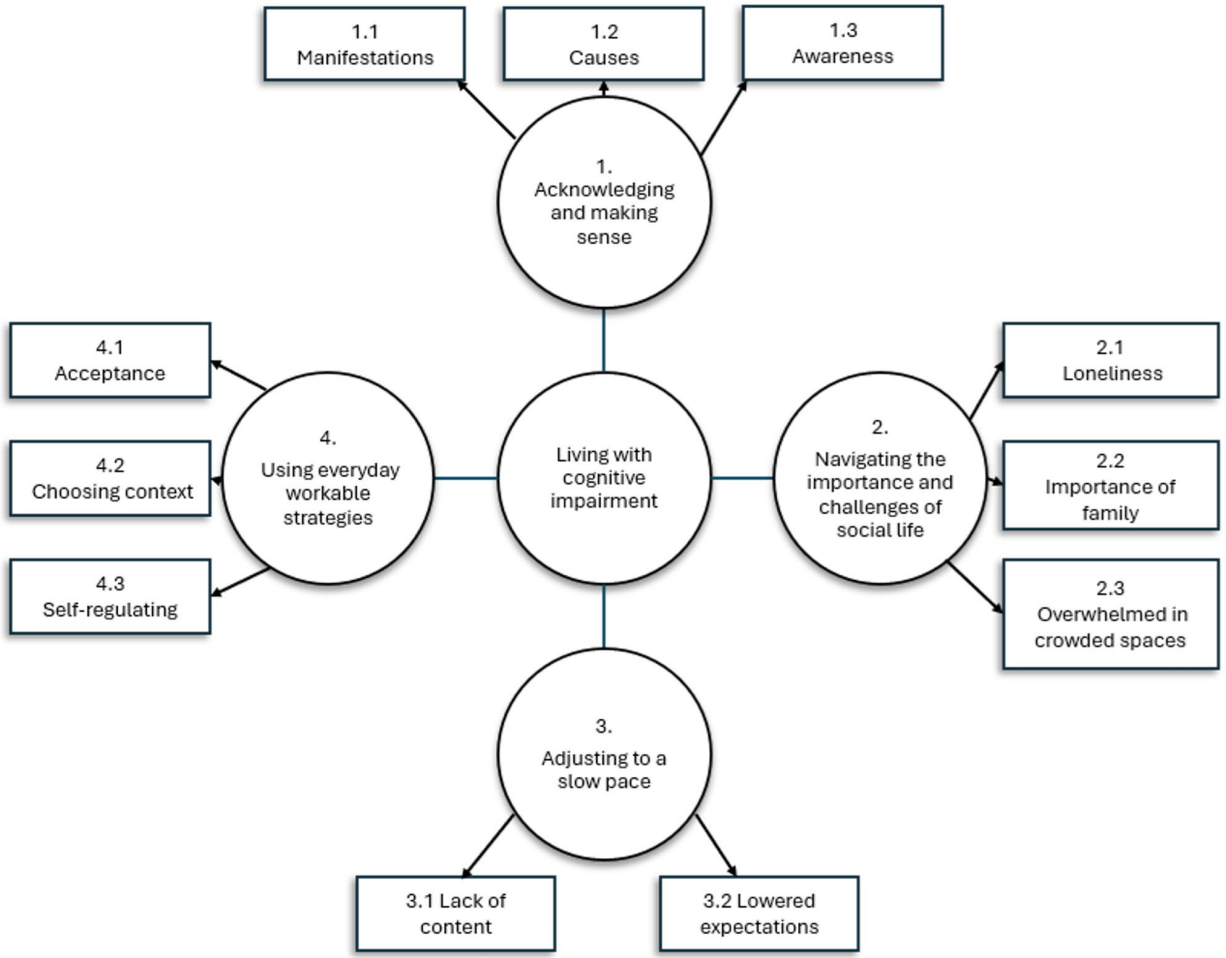
Thematic map. The four overarching themes relate to the central question of what it is like to live with psychosis and cognitive impairment

The analysis resulted in four overarching themes (see Fig. 1). The first theme, *Acknowledging and making sense*, comprised three subthemes: *Manifestations*, *Causes* and *Awareness*. The second theme, *Navigating the importance and challenges of social life*, included the subthemes: *Loneliness*,* Importance of family* and *Overwhelmed in crowded situations*. The third theme, *Adjusting to a slow pace*, comprised two subthemes: *Lack of content and Lowered expectations*. The fourth and final theme, *Using everyday workable strategies*, consisted of the subthemes *Acceptance*, *Choosing context*, and *Self-regulation.*

### Theme 1: Acknowledging and making sense

This overarching theme captures how participants described and made sense of their cognitive difficulties, with a focus on their understanding of the manifestations, perceived causes, and awareness of these challenges. It reflects a process of recognizing, interpreting and contextualizing the cognitive impairments they experienced.

#### Manifestations

This subtheme focuses on how cognitive difficulties took shape in participants’ everyday lives, focusing on the specific experiential domains where these problems surfaced. Rather than exploring how participants interpreted or made sense of these difficulties, the subtheme describes the concrete forms in which cognitive strain appeared. These difficulties ranged from more circumscribed problems, such as forgetfulness or slowed thinking, to broader difficulties that influenced participation in everyday activities. For example, one participant experienced difficulties in following social interactions:

If I’m in a group of people, for example, everyone is talking, it feels like I’m not really keeping up. It’s the same thing when I watch TV, I watch a lot of the news /…/ and I just feel like I can’t keep up with what they’re talking about. (P1)

A different perspective came from a participant who said that he did not experience cognitive difficulties in his everyday life. It was only when he had to solve “more complex problems,” as he put it, that it got difficult to control his thoughts:

Like… I’m trying to figure out how to solve something at work, these cables or whatever, and it’s like I see it but I don’t understand anything, and it just gets all foggy. It’s like my thoughts don’t go anywhere. (P8)

Performing the neuropsychological test in this study had made one participant feel at unease, something he said also happened in situations with a lot of stimuli:

It became so much and so intense when I did that test, so then I felt a bit spooky, as I usually say, when I left here /…/ Maybe it was about getting into a lot of concentration, you know, wanting to perform and stuff like that /…/ it’s when I have a feeling of unease, so to speak, like something could happen. (P7)

#### Causes

Participants described several different explanations for their cognitive difficulties. One type of explanation concerned the influence of psychotic symptoms. In one case, psychotic thoughts were described as taking up so much mental space that they disrupted attention and made it hard to focus on external information:

I was thinking about very strange things. Very psychotic thoughts. They took up so much space in my mind that it was hard to focus on what others were saying /…/ Once you had processed the information, it was hard to make anything meaningful out of it in your head. Instead, you thought it was a red thread /…/ Like it was part of your mission to read that. (P4)

Another participant linked her concentration and memory problems to experiencing her surroundings as frightening, describing how worry about being harmed intruded on her thoughts:

You kind of feel like you’re experiencing your surroundings as scary, like you might think someone is after you /…/ You feel like you need to escape from a situation /…/ it really affects your concentration and memory, and being able to keep things in your head. (P5)

A different kind of explanation concerned medication effects. One participant described how his partner noticed that he seemed to think more clearly when the medication was wearing off:

It’s usually like this, my girlfriend notices that I’m not on my medication. It’s not a bad thing, but she can tell that I understand things more. (P8)

#### Awareness

Participants varied in how clearly they were able to notice, articulate, and make sense of their cognitive difficulties. In this subtheme, awareness refers not to clinical insights in a diagnostic sense, but to participants’ ability to recognize and articulate their cognitive challenges in everyday life. Some participants described their challenges but struggled to specify their nature or explain them to others. One man attributed his difficulties to past psychotic episodes, but his explanation suggested a more general sense of decline rather than a clear understanding of which abilities were affected:

I’m not really in the game anymore, I think it’s the medication. Or maybe I’ve had too many psychoses, so eventually it leads to brain damage if you stay in that state all the time. (P6)

For others, the challenge was communicating their cognitive difficulties and how they affected them, even when they sensed something was wrong:

No, it’s hard to explain. I haven’t even managed to explain it fully to my family, they don’t really understand it. (P7)

By contrast, one participant seemed to have a higher degree of awareness. She actively engaged with the cognitive test feedback and reflected on how it aligned with her everyday functioning:

I just did a ChatGPT search for what visuospatial means, and it said things like reading maps, for example. I’m good at that kind of thing, like spatial stuff. I have a good sense of direction and all that, but then I’m really slow in my thoughts on certain things. (P1)

### Theme 2: Navigating the importance and challenges of social life

This overarching theme reflects how participants described the social aspects of their everyday lives, particularly the challenges they faced in social interactions. Cognitive difficulties, such as trouble keeping up in conversations, were commonly described and often contributed to feelings of loneliness and a reliance on family as primary social contacts. Some also described being restricted in their social engagements due to feeling overwhelmed by sensory input.

#### Loneliness

Several participants reflected on a desire to see other people more often, yet described cognitive challenges that made social interaction difficult. These challenges often contributed indirectly to experiences of loneliness. For some, television became a substitute for social contact:

I live alone. They’re my company. I don’t have a pet or anything… it’s really important to have that on, they have become my friends, those TV-characters. (P1)

One of the cases reflected on how he had changed over the years, becoming more introverted and finding it more difficult to understand humor and keep up in social interactions:

Now one just sits here and almost daydream these days, like… yeah… one has become more introverted in a way. (P6)

A further person expressed a wish to be more outgoing but explained how the sensory inputs became overwhelming when he was in social settings with a lot of people, which hindered his social engagement:

So that’s why I’m a bit scared of it when there are a lot of gatherings and stuff. I’d really like to go out to bars more to meet people and maybe flirt a little, but it’s a bit sensitive, so yeah. (P7)

#### Importance of family

Several participants emphasized the importance of support from family members. Spending time with family was often described as less demanding than interacting with friends or engaging in romantic relationships. For some, the family played a central role in their daily life:

My siblings call at least once a week. My parents call almost every day. (P4)

Several participants noted that family support ranged from concrete practical assistance, such as help with grocery shopping or reminders to complete tasks, to the provision of social connection and companionship. For one participant, having a family had been central to his recovery process. Although he experienced cognitive difficulties, he had been able to obtain and maintain employment, which he felt was largely due to the structure and activity that family life provided:

You know, a lot of this, when I entered family life, has made my mental health better. When I met my girlfriend and started a family, a lot of it is about staying busy. I’m so busy that I don’t need to talk about depression for days or for hours. (P8)

#### Overwhelmed in crowded spaces

As a barrier to social engagement, several participants described feeling overwhelmed by stimuli in environments with many people. One participant described the experience as follows:

It feels like the impressions become stronger, you’re not relaxed, almost like getting a fever in your head. (P7)

One man described social interactions as important, but explained that he felt exhausted even after something as simple as a walk to the local store:

It drains a lot of energy. Like when I go out on the street now, to a store, I’m completely exhausted afterwards /…/ Because I’m keeping up the mask, socially. I’m constantly looking around and having to make calculations: what’s happening now, what should I do now, how will this go, who is that, what does he want? (P4)

A similar tendency to be excessively affected by the surrounding environment was also reported. This diverted focus from what the participant was doing and consumed a great deal of energy:

Then you can feel pretty scattered, you know, and be quite influenced by your surroundings, maybe who is coming in and out of the café, and like what’s going on outside the café, and have trouble focusing on what they’re saying and responding appropriately, and things like that. So I’d say it can take a lot of energy. (P5)

### Theme 3: Adjusting to a slow pace

This theme captures participants’ experiences of a slow, often passive, daily rhythm. Several described lives with little structure and few planned activities. While some expressed a longing to be more active, many appeared to have adjusted by lowering their expectations and accepting a day-to-day existence with limited stimulation and few meaningful activities.

#### Lack of content

A sense of emptiness in daily life was a recurring experience. Several participants had few scheduled commitments or appointments and spent most of their time at home:

Yeah… yeah, I mostly stay at home. Mostly at home, but I do go to the store to buy bread and stuff like that. (P3)

There appeared to be a recurring difficulty in filling the days with meaningful activity. For some individuals this was a problem, causing boredom:

Yeah, it’s boring, it’s really boring. You just sit there squirming and wondering what to do, like… /…/ I usually turn on the TV so it gets better. (P6)

Occasionally, a more accepting attitude towards days with less activities was adopted, as by this man:

I’ve started to accept that life is very simple. There are very few things I do every day. (P4)

Even though he acknowledged doing very little each day, he expressed pride in the progress he had made. Now he could appreciate even the little things that he did, like brushing his teeth on a daily basis, something he had not done before.

#### Lowered expectations

A tendency to lower expectations regarding what a typical day might entail also emerged. One participant noted that he rarely had any appointments or obligations. In response to the question of whether an ordinary day usually turned out as expected, he replied:

Yeah… yeah… I don’t really have high expectations that it will turn into anything. (P2)

Another participant acknowledged that, from an outside perspective, his life might appear uneventful, yet he expressed a sense of acceptance—even though he wished things were different:

I hesitate a bit to go down to town, so to speak, so many people think I lead a boring life, but what can you do, you just have to accept it. (P7)

Yet another participant seemed to have adjusted his expectations due to his mental health challenges. While he was content with his current level of activity, he still expressed a desire to do more:

I’m satisfied with what I do considering I’m sick, but I would like to do much more. I would like to work, I would like to exercise. (P4)

### Theme 4: Using everyday workable strategies

This overarching theme concerns the ways in which participants managed their difficulties—an area they were specifically asked about during the interview. They reported a wide range of coping strategies, from specific behavioral adjustments to broader attitudes toward life. For example, one woman mentioned keeping as few belongings as possible, to make it easier to stay organized. Another participant emphasized the importance of facing one’s fears. However, some also noted that, at times, no effective strategies could be found to manage the challenges they faced.

#### Acceptance

Some participants reflected on the personal work they had done to adopt an attitude of acceptance. Rather than trying to eliminate their impairments or avoid certain situations, they focused on becoming more accepting of their limitations. One participant described how, before his first psychotic episode, he had been quick in both reading and writing. Now, several years later, he found himself much slower:

I try to avoid thinking about that it’s a big weakness /…/ So, if you’re sitting there trying to write something simple and it takes two hours, you get frustrated. So my way of dealing with it is just not thinking about how bad it is. (P4)

For him, accepting his current state—and recognizing the progress he had made—was key, even if he had not regained his former speed. Another participant described experiencing difficulty in following group conversations, which used to trigger anxiety in social settings such as parties. With the help of anxiety-reducing medications, she had become more accepting of her limitations and no longer avoided these situations, even though the difficulties following conversations remained:

If I’m invited to a party or something like that. Before, I could just say no, but now I go. And it feels good /…/ No, I just sit quietly and don’t say much, or laugh a little, you know, look a bit silly. (P1)

#### Choosing context

Several participants described how they managed their difficulties by selecting environments better suited to their needs. This often involved consciously avoiding certain situations and seeking out settings where they could navigate more comfortably. For example, one woman described discomfort in crowded department stores and explained how she adjusted her routines accordingly:

I avoid the times when it’s the most crowded /…/ I go either early or late. That’s when there are usually fewer people. (P9)

The same participant said that watching shorter video clips was important to her. She had tried watching television but found it too difficult. By changing format, she was still able to enjoy her interest:

They have reels that you can watch, short videos. I can concentrate on those /…/ But I can’t concentrate on the TV. It’s too long. (P9)

Many participants expressed discomfort in large groups. One man shared that he avoided going into town because it felt too crowded. Although he still had social needs, he found they were best met in quieter environments:

I don’t like going downtown. I find it stressful, there are too many people /…/ It usually goes fine if you hang out with one or two people like that. That’s not too stressful. (P6)

#### Self-regulating

Some participants described personal strategies they used to regulate themselves in challenging situations. Several reported feeling discomfort when overwhelmed by sensory stimuli and described methods they had developed to manage these experiences. One woman explained how she used breathing techniques to calm herself in busy environments:

If you’re in busy environments, you can try to focus on your breathing and things like that. That can help calm you down a bit. (P5)

Another participant said he often challenged himself to go into town despite finding it stressful. He had developed a clear coping strategy, which involved returning to a familiar and safe space to recover:

Then I usually go home, pull down the curtains, take my medicine, and then just wait it out until it gets better again. (P7)

One participant described difficulties with controlling intrusive or repetitive thoughts, referring to them as “triggers.” Through cognitive strategies, he had learned to engage with thoughts without becoming overwhelmed by them:

There is a quote by Aristotle saying that you should be able to entertain a thought without accepting it. That’s kind of what I do. I’ve focused a lot on distancing myself from thoughts. (P4)

## Discussion

This study used a qualitative methodology to gain a deeper understanding of how cognitive impairments affect the everyday life of people with psychosis, in order to inform the development of supportive interventions. Participants’ reflections were summarized into four overarching themes: (1) Acknowledging and making sense; (2) Navigating the importance and challenges of social life; (3) Adjusting to a slow pace; (4) Using everyday workable strategies.

### Acknowledging and making sense

This first theme reflects the well-documented heterogeneity of cognitive difficulties in psychotic disorders [5, 42]. What the present study adds is insight into how these difficulties were experienced in everyday life. Rather than neatly aligning with neuropsychological domains such as working memory or processing speed, participants described cognitive strain as emerging within specific experiential situations. They reported difficulties when following conversations, managing complex tasks at work or handling sensory and social demands. These accounts suggest that the experiential contexts in which cognitive challenges arise do not map directly onto the constructs assessed by standardized cognitive tests. This highlights a gap between clinically defined domains and the forms in which cognitive difficulties become functionally disruptive to the individual.

Participants also offered differing explanations for the origins of their difficulties. Some attributed cognitive strain to antipsychotic medication, while others linked it to psychotic symptoms or long-term illness. Prior literature indicates that cognitive impairments are largely distinct from psychotic symptoms [43–45], and to date we do not know whether antipsychotic medication helps or hinders cognitive functioning [21]. In samples such as ours, where most participants had long illness durations and ongoing antipsychotic treatment, disentangling these influences is methodologically challenging. Rather than resolving questions of causality, the findings point to the importance of understanding how individuals interpret the origins of their difficulties, as these interpretations may shape self-understanding and engagement with treatment.

Participants also varied markedly in their awareness of these difficulties. While all recognized some form of cognitive strain, the depth of this recognition ranged from a diffuse sense that “something was not working” to more differentiated accounts of strengths and weaknesses. Previous research has linked awareness of cognitive impairment to functional outcomes and readiness to engage in cognitive remediation [46, 47]. These findings suggest that when manifestations, perceived causes, and level of awareness are taken together, they form the individual’s own conceptualization of their cognitive challenges, a conceptualization that may differ substantially from clinician- or test-based models. Recognizing and working with this perspective may therefore be important for increasing the acceptability of cognitive interventions and for helping individuals understand how such treatments relate to the difficulties they experience in daily life.

### Navigating the importance and challenges of social life

Participants’ accounts reflected a desire for social connection alongside substantial barriers to participating in social life. Loneliness is well documented in psychosis [48], yet the mechanisms underlying this experience remain insufficiently understood [49]. The present findings suggest that cognitive challenges may contribute to these experiences, particularly when participants struggled to follow conversations, keep up with multiple speakers, or manage overwhelming sensory input.

At face value, these challenges may resemble impairments in social cognition, yet they do not fully align with how social cognition is conceptualized in the literature. Rather than difficulties interpreting others’ intentions or emotions [50, 51], participants mainly experienced social situations as intense, often feeling “overwhelmed by stimuli”. This phenomenon, frequently studied under the term sensory gating, refers to atypical filtering of irrelevant information [52] and has primarily been examined in laboratory settings using specialized procedures rather than standard neuropsychological tests [53, 54]. Taken together, the present findings indicate that for some individuals, barriers to social participation may arise less from difficulties with social cognition (i.e., interpreting others’ intentions or emotions) and more from cognitive overload during interactions, reflecting the real-time processing demands of complex social situations.

In the absence of broader social exchange, interactions with family served as an especially important source of contact, consistent with evidence on the substantial but often invisible role of informal caregivers [55]. One possibility is that family members can adjust expectations and interactional pace in ways that reduce cognitive demands. Understanding these interactional conditions may help inform how social community spaces could be designed or adapted to better accommodate individuals who experience cognitive overload in social situations. This is particularly important given that not all individuals have access to supportive family networks, and over-reliance on family risks placing additional burden on caregivers.

These findings, together with the context-specific ways cognitive strain was described in the first theme, highlight that cognitive challenges in psychosis manifest in ways that may not be captured by standard tests, yet significantly impact daily social functioning.

### Adjusting to a slow pace

Many participants described a sense of emptiness in their daily lives, often marked by a lack of meaningful content. These accounts resonate with evidence showing that employment is associated with higher life satisfaction and broader social networks among individuals with psychotic spectrum disorders [56–58]. Importantly, it does not appear to be formal employment itself that matters most, but rather access to a structured, work-like occupation [59]. Difficulties engaging in such activities may therefore amplify isolation and reduce quality of life, underscoring the need to better understand the specific cognitive barriers to participation.

A related theme was participants’ lowered expectations for daily life. While this may reflect negative symptoms such as reduced motivation and interest [60], it can also be interpreted through the framework of defeatist performance beliefs (DPB). DPB refers to negative beliefs about one’s ability to perform tasks and has been proposed as a mediating process linking cognitive impairment to reduced motivation [61]. Cognitive difficulties may lead to discouraging experiences that accumulate over time, reinforcing DPB and contributing to withdrawal and passivity [62]. Targeting these beliefs may therefore represent a promising avenue for interventions aimed at improving functioning and quality of life [63, 64].

### Using everyday workable strategies

Participants described a range of strategies for managing their cognitive difficulties that focused less on compensating for deficits and more on adjusting daily activities to fit their current capacities. Several participants described an accepting stance toward their challenges, which appeared to reduce the risk of developing defeatist beliefs. This resonates with principles of Acceptance and Commitment Therapy for psychosis (ACTp) [65], which aims not to remove symptoms but to transform the individual’s relationship to them. Although ACTp was not developed to specifically target cognitive difficulties, emerging evidence from conditions other than psychosis suggests that ACT may positively influence aspects of cognitive functioning [66]. This may represent an interesting avenue for coping with cognitive difficulties in psychosis, where acceptance can support continued engagement in valued activities despite ongoing challenges.

Another strategy involved actively choosing environments that minimized cognitive strain, such as quieter places or situations involving fewer people. Although rarely emphasized in reviews of how individuals with psychosis manage cognitive difficulties [67], this form of environmental tailoring can be understood as a practical expression of self-awareness. By aligning situational demands with perceived capacities, individuals created conditions in which they could function more effectively and with less distress.

In relation to the first theme, which highlighted variations in awareness of cognitive challenges, the present findings suggest that increased awareness may facilitate the development of practical strategies for navigating everyday life. Previous research has highlighted the importance of awareness of cognitive difficulties for engagement in treatment programs [47, 68]. The present findings extend this perspective by indicating that greater awareness may also support the generation of self-developed strategies, highlighting the potential value of supporting awareness as a foundation for practical coping in daily life.

### Limitations and strengths

The inclusion criterion—requiring only one out of five RBANS index scores to fall below average (< 85 points)—might be viewed as relatively lenient compared to other studies, which have required more substantial or multiple impairments. However, a more stringent threshold would likely have made recruitment considerably more difficult, as significant cognitive impairments can limit individuals’ ability to participate in research. In fact, one participant who had initially expressed strong motivation to take part became overwhelmed after only one subtest and chose to withdraw. This highlights a methodological concern: cognitive tests may act as gatekeepers, unintentionally excluding individuals with the most severe difficulties. Future studies may benefit from exploring alternative ways to assess cognitive impairment for inclusion purposes. While some participants in the present study had relatively mild impairments, the sample still exhibited meaningful variability.

The central role of the research leader in all stages of the study, from assessment to interviews and analysis, poses a risk of bias. However, this sustained involvement also enabled multiple interactions with each participant, likely contributing to trust and openness during the interviews. To further mitigate potential bias, the co-authors were actively involved in the interpretation of results, allowing for triangulation and critical reflection. Another limitation is that current clinical symptoms were not assessed. Prior research shows that individuals with psychosis often struggle to distinguish cognitive from other psychiatric symptoms [35], complicating efforts to attribute daily life difficulties to specific symptom domains. However, this ambiguity may itself be an important finding, illustrating how these experiences often merge in real-world contexts. Another limitation concerns the sample’s limited sociocultural diversity. In line with Swedish research practices, race and ethnicity were not collected, which, together with the small sample size, restricts the transferability of the findings to more diverse populations. Recruitment was also limited to outpatient services, meaning that individuals in more acute illness phases or other care settings were not represented. Future studies may benefit from including participants across a wider range of clinical and cultural contexts.

## Conclusions

This study highlights the heterogeneity in manifestations of cognitive impairment, as well as variations in individuals’ awareness of these challenges. These findings point to the need for individually tailored interventions, both to address specific cognitive difficulties and to enhance awareness as a means of strengthening coping and treatment engagement. The open-ended interview approach revealed difficulties with sensory gating, illustrating the often-overlooked impact of sensory overload on daily functioning. This points to the value of quiet spaces and suggests that community and clinical environments should be designed with sensory regulation in mind. Furthermore, participants’ lived experiences resonate with core principles of ACT for psychosis (ACTp), including acceptance, behavioral activation, and values-based coping. This indicates that ACTp-informed approaches may offer a clinically relevant framework for developing interventions that support psychological flexibility and help counter the development of defeatist performance beliefs.

### Implications for clinicians and future research

For clinical practice, the findings indicate that barriers to accessing cognitive interventions may partly stem from limited awareness of cognitive difficulties. Several participants struggled to articulate or understand their challenges, which may reduce acceptability and motivation for treatments such as cognitive remediation. Early, collaborative conversations about when and how these difficulties occur may help create a shared language, clarify treatment relevance, and lower barriers to engagement. For future research, the results suggest a need to develop and evaluate interventions that aim to enhance individuals’ awareness of cognitive impairment. Greater attention should also be given to sensory processing difficulties, which may contribute to functional challenges. With regard to existing treatments such as cognitive remediation, it may be valuable to explore differentiated treatment pathways. Individuals with limited awareness could initially be gently supported in reflecting on their difficulties and developing greater awareness, while others may benefit more from acceptance-based approaches or from more direct cognitive training. Such tailoring could help identify what works best for whom and improve both engagement and outcomes.

## Data Availability

Qualitative data are not publicly available due to confidentiality, but can be discussed with the research leader (TL) on reasonable request.

## Abbreviations

ACTp: Acceptance and Commitment Therapy for psychosis
DPB: Defeatist Performance Beliefs
RBANS: Repeatable Battery for the Assessment of Neuropsychological Status
